# A Review on Antimicrobial Packaging from Biodegradable Polymer Composites

**DOI:** 10.3390/polym14010174

**Published:** 2022-01-02

**Authors:** Siti Hasnah Kamarudin, Marwah Rayung, Falah Abu, So’bah Ahmad, Fatirah Fadil, Azrena Abdul Karim, Mohd Nurazzi Norizan, Norshahida Sarifuddin, Mohd Shaiful Zaidi Mat Desa, Mohd Salahuddin Mohd Basri, Hayati Samsudin, Luqman Chuah Abdullah

**Affiliations:** 1 School of Industrial Technology, Faculty of Applied Sciences, Uitm Shah Alam, Shah Alam 40450, Malaysia; falah@uitm.edu.my (F.A.); sobah@uitm.edu.my (S.A.); fatirahfadil@uitm.edu.my (F.F.); rena184@uitm.edu.my (A.A.K.); 2 Department of Chemistry, Faculty of Science and Technology, Universiti Putra Malaysia, Serdang 43400, Malaysia; marwahrayung@yahoo.com; 3 Centre for Defence Foundation Studies, Universiti Pertahanan Nasional Malaysia (UPNM), Kem Perdana Sungai Besi, Kuala Lumpur 57000, Malaysia; mohd.nurazzi@gmail.com; 4 Department of Manufacturing and Materials Engineering, International Islamic University Malaysia, Jalan Gombak, Kuala Lumpur 53100, Malaysia; norshahida@iium.edu.my; 5 Faculty of Chemical Engineering Technology and Process, Universiti Malaysia Pahang, Lebuhraya Tun Razak, Gambang 26300, Malaysia; shaiful@ump.edu.my; 6 Department of Process and Food Engineering, Faculty of Engineering, Universiti Putra Malaysia, Serdang 43400, Malaysia; salahuddin@upm.edu.my; 7 Food Technology Division, School of Industrial Technology, Universiti Sains Malaysia, Pulau Pinang 11800, Malaysia; hayatis@usm.my; 8 Department of Chemical and Environmental Engineering, Faculty of Engineering, Universiti Putra Malaysia, Serdang 43400, Malaysia; chuah@upm.edu.my

**Keywords:** antimicrobial packaging, biodegradable, natural fibre, polymer composite, sustainable

## Abstract

The development of antimicrobial packaging has been growing rapidly due to an increase in awareness and demands for sustainable active packaging that could preserve the quality and prolong the shelf life of foods and products. The addition of highly efficient antibacterial nanoparticles, antifungals, and antioxidants to biodegradable and environmentally friendly green polymers has become a significant advancement trend for the packaging evolution. Impregnation of antimicrobial agents into the packaging film is essential for impeding or destroying the pathogenic microorganisms causing food illness and deterioration. Higher safety and quality as well as an extended shelf life of sustainable active packaging desired by the industry are further enhanced by applying the different types of antimicrobial packaging systems. Antimicrobial packaging not only can offer a wide range of advantages, but also preserves the environment through usage of renewable and biodegradable polymers instead of common synthetic polymers, thus reducing plastic pollution generated by humankind. This review intended to provide a summary of current trends and applications of antimicrobial, biodegradable films in the packaging industry as well as the innovation of nanotechnology to increase efficiency of novel, bio-based packaging systems.

## 1. Introduction 

Packaging is a billion global industry and plays a significant role for essential items for consumer goods ranging from basic chemicals to household and personal care products, drinks, foods, medical devices, and much more. The value of the packaging industry is highly expanding due to competitiveness in making commodities and luxury packaging. To date, the applications of plastics in the packaging sectors have been increasing at a fast speed due to their benefits of being commercially low cost and possessing intrinsic characteristics of plastic films in packaging industries. The most frequent plastic films used in the development of the packaging industry include polypropylene (PP), low-density polyethylene (LDPE), linear low-density polyethylene (LLDPE), poly(vinyl chloride) (PVC), and poly(ethylene terephthalate) (PET). The unique properties of plastics such as low cost and superior processability and having good barriers, mechanical properties, good sealing characteristics, and high transparency make them a favorable material [1]. In addition, they can be totally recycled and are lightweight alternatives to traditional, non-recyclable materials due to their super functionality [2,3,4]. Despite all the listed usefulness and benefits, the use of plastics as base materials in the packaging system suffered from the limitations of the materials themselves, such as the harmful chemicals and waste that packaging leaves behind. The wide usage of plastic packaging has caused serious plastic waste disposal problems, which, in turn, create massive environmental pollution [5]. In 2018, the World Wildlife Fund also reported that China, Indonesia, Malaysia, the Philippines, Thailand, and Vietnam contributed around 60% of the estimated 8 million tonnes of plastic that enter the world’s oceans every year [6]. This threat to the environment is basically due to the significant level of highly toxic emissions, composting management issues, and alteration in carbon dioxide cycle [7]. Furthermore, disposed packaging plastics in many countries are rarely recycled due to technical issues and socio-economic constraints. For example, in China, there is only about 20% of recycled plastic waste as compared with 1 million tons of plastic generated [8]. Moreover, a huge proportion of the used plastic materials is either deposited in landfills or contributes to litter everywhere, surrounding the environment, which ends up putting stress and strain on the environmental balance. The alternative way to minimize the waste contributed by plastic is to use compounds from nature. Therefore, this phenomenon has stimulated the attention of many researchers to develop sustainable, active packaging material [9]. Therefore, the design of the packaging should consider not only shelf-life, cost, and protection, but also user-friendliness and environmental sustainability [10].

**Figure 1:**
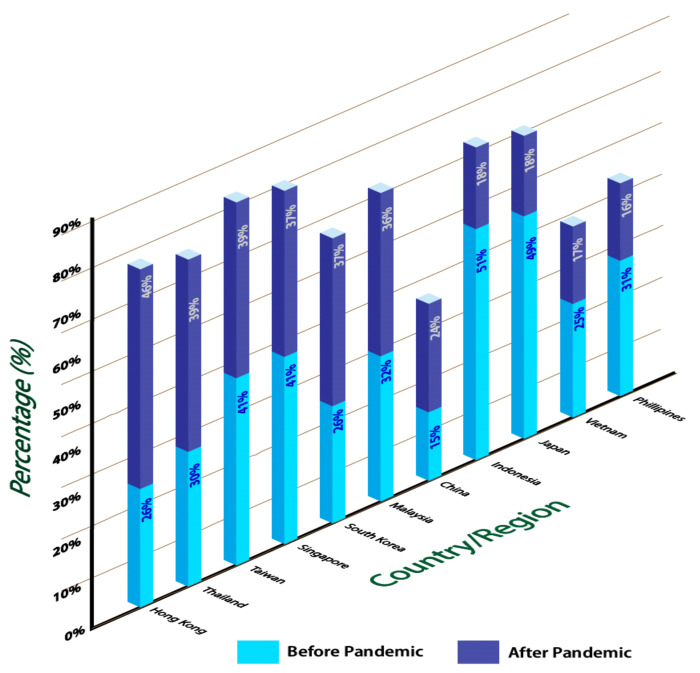
COVID-19’s impact on consumers ordering food, APAC 2020, by country or region. Modified from [11].

The consumption of food packaging was said to have increased during this pandemic (Figure 1) [11]. A comparison of different regions shows that the consumption of food packaging before and after the Covid-19 pandemic vary strongly. Apparently, Indonesia has contributed to a large amount of food packaging consumption before the pandemic caused by Covid-19. During the pandemic, Hong Kong passed as the highest region consuming food packaging. Because of the pandemic, there is a high concern regarding the hygiene and safety aspects by customers. Most people have resorted to their last option of buying bulk stocks of groceries or having their meals taken away. According to the Agriculture and Horticulture Development Board (AHDB) in its 2020 article on *‘Takeaway food performance during Covid-19’*, the pandemic effect has urged people to switch from dine-in to takeaway-delivery due to social distancing recommendations.

One of the important safety aspects related to food packaging is its influence towards the microbial shelf life of food. In the environment where we live, there are millions of microbes, most of which are not visible to the human eye. The microbes, such as viruses and bacteria, have a very simple composition and replicate very quickly. For instance, a single bacteria can generate up to 500 new bacteria within 3 h through binary fission. Some of these bacteria and viruses may cause infections, ranging from mild to deadly diseases. Throughout human existence, dangerous microbes have been a source of horrifying epidemics such as plague, cholera, tuberculosis, etc. Although they are invisible, microbes continue to cause health problems, especially to the respiratory, digestive, and nervous systems. The types of diseases found today have been extremely difficult to prevent and cure due to high levels of antimicrobial resistance. Microbes can be transmitted in the following ways: (1) Coughing and sneezing, (2) Breathing contaminated air, (3) Contact with infected people by shaking hands, and (4) Contact with the infected objects or contaminated surfaces, water, or food. The threat posed by bacteria has inspired numerous researchers to research and develop unique antimicrobial plastic packaging for farm, food, and cosmetics. 

**Figure 2:**
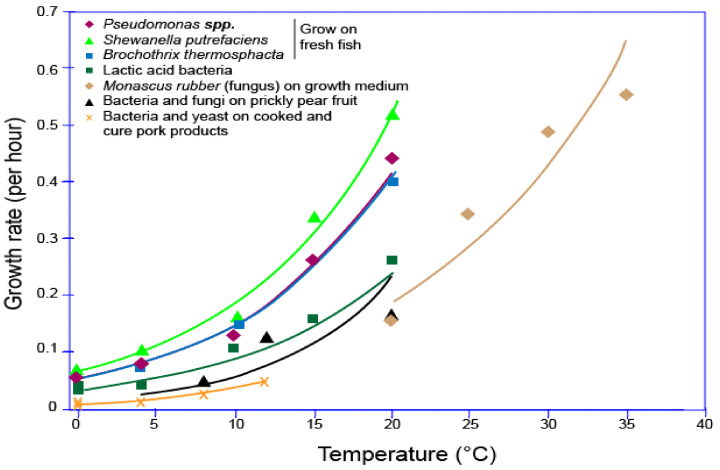
Growth rate of various types of microbes depending on the time. Modified from [16].

The reactions of bacteria, enzymes, molds, and some microorganisms towards the surrounding humidity and temperature on different types of foods also contribute to food spoilage in the food packaging [12,13,14,15], as displayed in Figure 2 [16]. Figure 2 shows that *Shewanella putrefaciens’* growth rate on fresh fish was the highest compared to Pseudomonas spp. and *Brochothrix thermosphacta*, which was around 0.5 per hour at 20 °C. On the other hand, bacteria and yeast on cooked and cured pork products showed the lowest growth rate, which was less than 0.1 per hour at around 12 °C. However, *Monascus ruber* (fungus) showed a unique growth rate, which started when the temperature was at 20 °C with less than 0.2 per hour and rose gradually to more than 0.6 per hour. Some researchers did their research on how to lower this carbon footprint [17,18]. 

There are a number of limitations in current packaging, which are non-sustainable production, legislation, cost pressures, and consumer education. Helping consumers to understand the importance of packaging, whether it is in food, drink, or medicine or giving economical access to products they need every day to make their life easier, safer, and more confident not only about the products they buy but the role of the packaging it is served in helps ensure those products maintain freshness, quality, and efficacy. Thus, poor barrier properties to water, vapor, and gases are the important critical issues in packaging. Fresh products like vegetables and fruits need to be packaged in an oxygen-permeable membrane environment, whereas processed products do not require much transfer. Another challenge faced by many producers is speed to market. A shorter research and development (R&D) process is needed for the development of packaging, which is around 9 months instead of the 12 to 18 months for the current packaging development cycle. Food waste reduction as well as new consumer experiences in new consumption occasions for the benefits of consumers and protecting the food quality are among the biggest challenges for current packaging right now. Additionally, bringing new, innovative products and at the same time maintaining sustainability goals and profitability goals both for consumers and the packaging company are the reforms that need to be made. The excessive growth of microorganisms because of contamination and temperature abuse, the high degrees of nutritional qualities to the oxidation, and laws of nutritional qualities to the interaction with extreme factors are among examples of food quality and safety issues.

Antimicrobial packaging was introduced to combat this problem so that the shelf-life storing of the food can be extended, reducing food waste [19,20]. Apparently, antimicrobial agents had been applied to be incorporated with the food packaging [21,22,23,24,25] The antimicrobial properties in antimicrobial agents have made them become suitable to be incorporated with food packaging [26,27,28]. According to Rhim et al., antimicrobial-function nanocomposites were found effective for minimizing the growth of contaminant pathogens that exist after the post-processing, extending food shelf-life and enhancing food protection [29].

The usage of green polymers together with nanoparticles such as silver nanoparticles (AgNPs) as an antimicrobial agent is common in food packaging industries [30,31] since the characteristics of silver nanoparticles are good enough to make them a widely used nanofiller for making packaging because of their antimicrobial properties. Fillers are substances that are applied to regular packaging products, typically in low percentages, to improve the performance of the original content. In a composite, it is basically the mixture of the regular packaging material and a filler [32]. From the collective reviews that were done, it can be summed up that those researchers mostly used silver nanoparticles (AgNPs) as their antimicrobial agent because of their effective antimicrobial performance, low toxicity, and high thermal stability, and they have continued to gain attention since. In addition, it is possible to significantly increase the stability and mechanical strength of poly-saccharide films by adding AgNPs [33]. Evidently, a few articles that have been published in the field of bio-composites and bio-nano composites agreed on the potent properties of silver nanoparticles in making antimicrobial food packaging with a longer shelf-life [31,34,35]. 

Even though biopolymers are environmentally friendly and considered as most fascinating packaging materials, the industrial applications are restricted due to several factors such as their oxygen/water vapor barriers, thermal resistance, and other mechanical properties associated with costs [36]. In order to encounter these challenges and urge the industrial applications of biopolymers for packaging materials, there is the requirement for advanced research to effectively improve their stability, quality, nutritional values, and microbial resistance. Moreover, the barrier properties need to be intensified. Biodegradable polymers containing starch/cellulose fibres are most likely to make a solid growth in applications. Numerous approaches for elevating the properties and performance of antimicrobial packaging materials, such as chemical and physical modifications, polymeric blending, and nanocomposites, have indicated a bright potential for many types of applications. 

Hence, more advanced research tools and huge investment are required to obtain fully sustainable materials with antimicrobial activity and effective alternatives for the existing ones. The enhancement of a moisture barrier and mechanical and other properties of biodegradable polymer will benefit the significant innovation in these packaging materials. Moreover, an increment in the use of biodegradable packaging must be intensified by more composting infrastructure. The development of specialised recycling procedures for these types of packaging should be considered. Despite all the advantages related to the use of silver nanoparticles with biodegradable polymers for sustainable packaging and a safer environment, several important constraints are minimizing the toxicity and environmental risks impacted from the packaging waste containing these nanoparticles.

Both functional and technical gaps have been the limitation barriers toward the development and applications of antimicrobial packaging materials in industries. Several limitations include vapor and air barriers, the stability of antimicrobial agents under processing conditions, and the low processability of bioplastics’ toxicity as well as the changes in mechanical properties of the packaging materials. Accordingly, further research work should be focused on filling the void linking the antimicrobial actions to microbial growth kinetics in the packaged foods in both lab and industrial approaches. Close collaboration between both academic and industrial players could be an effective alternative to filling the gap between commercial aspects and research. Synergism and blending of nanocomposites would be the core tools as the useful strategies for improving antimicrobial performances for improving antimicrobial packaging and preventing some of the limits encountered during activity. This would contribute to the initial essays on the research and development of antimicrobials’ packaging.

In addition, a forecast of market demand shows that the estimated global market growth for antimicrobial packaging was exponentially increased, as indicated from a growing Compound Annual Growth Rate (CAGR) value from 2020 until 2024. The contribution in the increasing economic value of these antimicrobial packaging products from biodegradable polymer composites is driven by the growing awareness of the consumers towards the consumption of sustainable and green packaging. Consumers are now consciously aware of the possible threat coming from synthetic food preservatives to human health, as some are potentially transformed into carcinogenic agents, thus indirectly helping in reducing the dependency on the consumption of synthetic preservatives. The use of these antimicrobial packagings from biodegradable polymer composites will be greatly beneficial in accelerating the transition towards preservative-free food products. The vast potential of antimicrobial packaging in sustaining the freshness of some selected highly perishable food including meat and poultry, seafood, fruits and vegetables, baked goods, and cheese and dairy-based products has contributed to the rise in the market demand, thus creating growth profitability for the players operating in the global market.

This review focused on the summary of current trends and applications of antimicrobial biodegradable films in the packaging industry as well as the innovation of nanotechnology to provide high efficiency of novel, bio-based packaging systems. For that reason, the influence of attractive product packaging plays an important role in the consumer purchasing decision. Most consumers are looking into new, added value possessed in the advanced packaging technology over the traditional packaging. The ideal antimicrobial packaging materials should be equipped with intelligent indicators’ technology to measure certain crucial conditions, such as temperature, pH value, and humidity, to show the degree of bacterial contamination developed in packaged food throughout its shelf life. Universal protocol standards are needed not only to evaluate their antimicrobial activity against common food-borne bacteria and maintaining food quality, but also to meet consumer sensory preferences. The alternative packaging asserts to perform similarly as conventional packaging in terms of achieving expected shelf life of food, durability, sealing strength, printability, and flexibility. The integration of these responsive technologies into food packaging will provide a massive impact in the food processing industries, to fulfil the growing demand for packaged, ready-to-eat foods that are distinguished as a primary driver of future packaging trends. 

## 2. Antimicrobial Packaging Agents

Antimicrobial packaging can be produced by the addition of antimicrobial agents such as chitosan and essential oil into the systems [37,38]. The incorporation of antimicrobial agents can be done by several techniques such as direct addition, encapsulation, coating, or grafting into or onto the matrix [39]. Meanwhile, polymeric materials are often used in conventional packaging systems. These polymers can be obtained from various sources and they can be classified based on their biodegradability. A current trend has been focusing on replacing non-biodegradable polymers to biodegradable polymers due to environmental concern, legislative rules, and consumer demands for green products. In view of this, the antimicrobial packaging has also been shifted to produce products from natural resources for both the host polymer and the antimicrobial agents. The types, advantages, and their limitations will be discussed in the following subtopic. 

**Figure 3:**
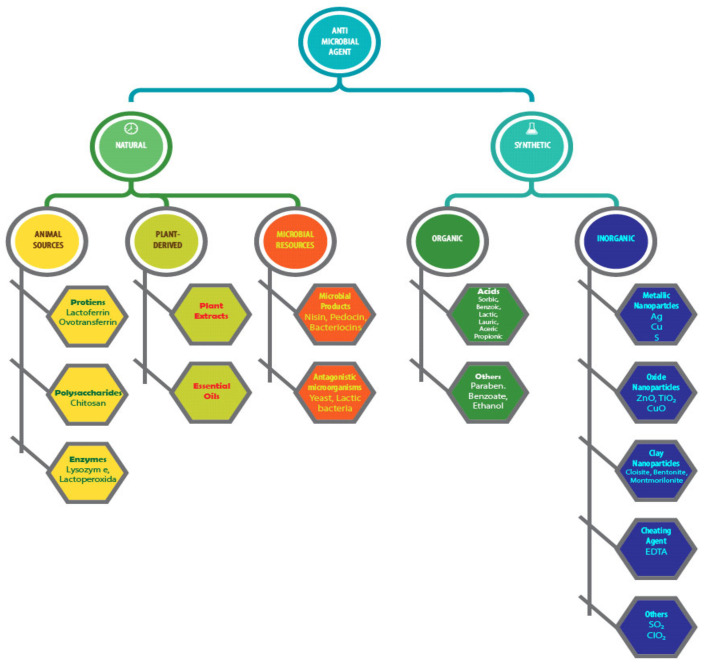
Classification of antimicrobial agents. Modified from [49].

Various types of antimicrobial agents have been investigated for their potential applications in the antimicrobial packaging, and some of the examples are presented in Figure 3. Each of the antimicrobial agents has a unique mechanism and reacts differently to different types of microorganisms. In this case, the types of antimicrobials sometimes set restrictions for their applications. In general, these compounds can be classified into synthetic and natural classes based on their sources and physiologies. The synthetic antimicrobial agents can be further categorized as organic and inorganic. The literature reports various types of synthetic antimicrobial agents such as metallic nanoparticles (Ag, Cu, S) [40], oxide nanoparticles (ZnO, TiO_2_, CuO) [41], clay nanoparticles (bentonite, cloisite, montmorilonitrile) [42], chelating agents [43], volatile compounds (SO_2_, ClO_2_, ethanol) [44], organics acids and their salts [45], etc. [25,46]. The type of antimicrobial agents selected may differ depending on for which application the packaging material is being used. Basically, synthetic organic compounds containing ethylene diamine tetraacetic acid (EDTA), parabens, fungicides, and other chemicals are the major antimicrobial compounds used in the food packaging industry [47]. In particular, metal and metal oxides are potential antibacterial agents, but issues concerning their long-term impact on the environment and human health remain unresolved. On the other hand, the use of silver-based antimicrobial packaging for food purposes is expected to grow and has been used in countries such as Japan and the United States [48]. 

**Figure 4:**
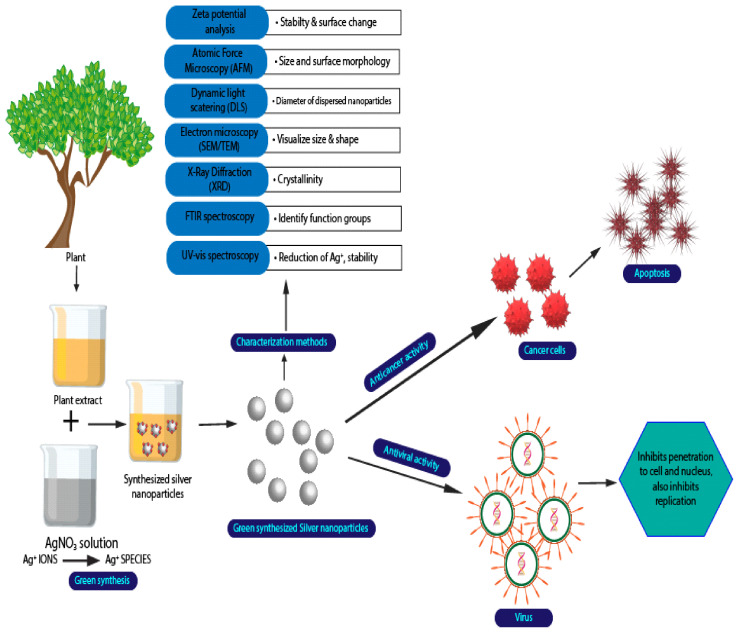
Biological synthesis of silver nanoparticles using plant extraction.

There were two ways of synthesizing the AgNPs in biological methods, which are intracellular or extracellular [50,51,52]. For the extracellular process, it involves trapping the metal ions on the outer surface of the cells. Then, it aims to reduce the silver in the presence of biomolecules. On the other hand, for the intracellular, the process takes place inside the microbial cells [53]. Extracellular synthesis was shown to be preferable in most studies compared to intracellular due to its simplicity, lower cost, and preferred large-scale production [51,53,54]. Additionally, some of the advantages of using this biological method are that it is environmentally friendly, does not produce any toxic residue, and is cost-effective [53,54,55,56,57,58,59]. Figure 4 shows the illustration of biological synthesis of silver nanoparticles using plant extraction.

**Figure 5:**
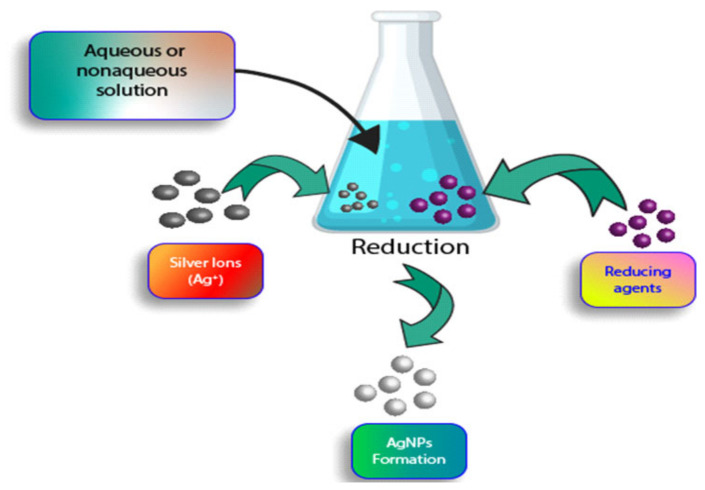
Chemical synthetization of silver nanoparticles through chemical reduction.

Next, chemical synthesis of silver nanoparticles was also reviewed [53,55,59]. In this method, chemical reduction was discussed [53,55]. Reducing, stabilizing, or capping agents were used in this method. The size distribution of the produced silver nanoparticles was stated to be influenced by these agents [57]. The simple equipment used and the convenience of the chemical reduction are the benefits of using this method [60]. Nonetheless, this method is not widely preferred due to the requirement of using harmful chemicals such as sodium citrate, borohydride, potassium bitartrate, and sodium dodecyl, which are very toxic [53,61]. Additionally, this method was mentioned to yield toxic by-products during the process [59]. Figure 5 shows chemical synthetization of silver nanoparticles. 

The positive attributes, such as being biocompatible, safe, and non-toxic to the environment, can be especially effective in food packaging, while posing no hazard to humans [62]. Natural antimicrobial agents come from various sources and they can be obtained from animals, plants, or microbial sources [63]. Some commonly used antimicrobial agents are from animal sources such as proteins (lactoferrin, ovotransferrin), enzymes (lysozyme, lactoperoxidase), and polysaccharides (chitosan). Additionally, microbial products such as nisin, pediocins, and bacteriocins are often used as antimicrobial agents in packaging. In a similar way, plant-derived antimicrobial agents are usually obtained in a form of essential oil or extract. Some of the most effective natural extracts are ginger, garlic, oregano, thyme, cinnamon, clove, coriander, and more. The presence of active compounds in these materials such as flavanols, terpenes, anthocyanins, phenolic acids, tannins, and stilbenes is responsible for the antimicrobial effects for specific microorganisms. Moreover, they could provide additional health benefits such as being nutritional supplements [48]. From this point of view, the use of antimicrobial agents from plant-derived sources could be an excellent choice especially for food packaging purposes. At present, the challenge related to the plant-derived antimicrobial compounds is due to their loss during high-temperature processing and reduced antimicrobial efficiency [64]. Other factors that restrict their application are the production cost and sometimes the strong aroma produced [63]. 

### 2.1. Polymeric Matrix Used in Antimicrobial Packaging

The selection of a polymeric material is dependent on the intended use on application and highly depends upon the properties of the polymer matrix [65]. Polymers such as poly(ethylene terephthalate) (PET), polyethylene (PE), poly(vinyl chloride) (PVC), polypropylene (PP), polystyrene (PS), and others have been investigated in this field. PVC is one of the examples used for polymers for packaging in the world. It has several advantages including flexibility, toughness, light weight, and ease of processing. Studies on PVC loaded with silver nanoparticles [66], zinc [67], and orange essential oil [68] have been reported. PET is another type of polymer that is useful and has the potential to be as good as PVC. This polymer has a good mechanical strength and toughness [69]. Further, PE is another widely used polymer. Low-density polyethylene (LDPE) is the cheapest among other polymers. Meanwhile, PP with high grade has a high melting point; thus, it is suitable for high-temperature packaging [47]. These materials are often used because of their good properties and relatively low cost [70]. Table 1 shows some examples of petroleum-based polymers that have been studied as a host with a variety of antimicrobial agents for packaging materials.

### 2.2. Antimicrobial Packaging from Bio-Based Polymers

**Figure 6:**
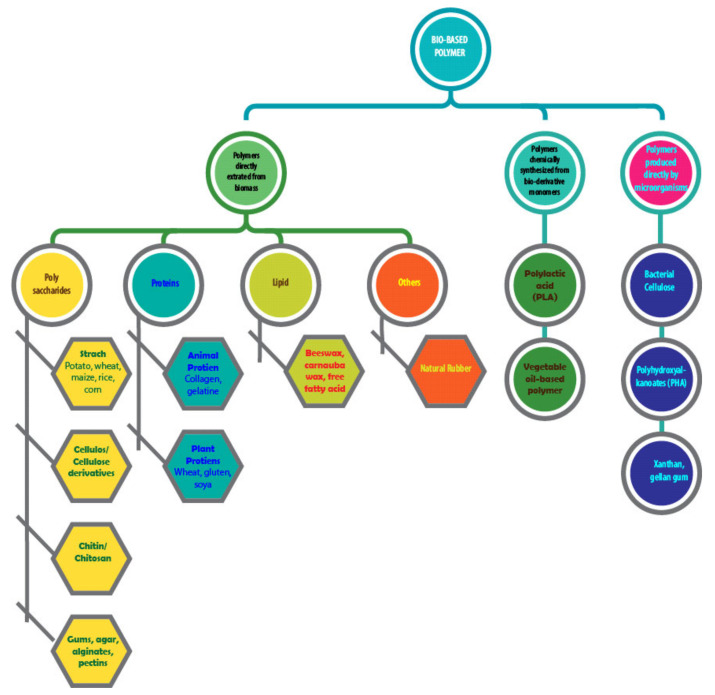
Classification of bio-based polymers based on their origin.

Despite the excellent performance of the petroleum-based polymers, they possess several limitations. One of the main constraints is their non-biodegradable properties that can cause short- and long-term pollution [69]. To address this issue, much attention has been given by the researchers and industries in developing antimicrobial packaging from bio-based polymers [82]. The use of bio-based polymers in place of traditional petroleum-based polymers could avoid the disposal problem and produce products that are environmentally friendly, safer, and non-toxic. Additionally, these materials give advantages in the sense that they are renewable and available abundantly in nature. The general classification of bio-based polymers is depicted in Figure 6. They can be broadly classified into three categories, which are (1) polymers directly extracted from biomass sources, (2) polymers chemically synthesized from bio-derived monomers, and (3) polymers produced directly by microorganisms [83]. Polymers such as poly(lactic acid) (PLA), starch, cellulose, and chitosan are gaining more favor in the antimicrobial packaging.

PLA is a type of biodegradable and renewable polymer that has been studied extensively for antimicrobial packaging. PLA is obtained from two major pathways: ring opening of lactide or direct polycondensation of lactic acid, a monomeric precursor derived from renewable resources. The monomer was produced from the fermentation process of sugar feedstock such as dextrose or chemical synthesis. Sugar feedstock can be obtained in two ways: firstly, directly from sources (sugar cane, sugar beets) or secondly, through conversion of starch from corn, potato, wheat, rice, or agricultural waste [70]. PLA has mechanical properties that are almost similar to commercial thermoplastics like PET, making it possible to be applied in a wide range of products [84]. Starch is a promising biodegradable and biocompatible polymer in the packaging industry. It is non-toxic and readily available. However, starch has a strong hydrophilic behavior, thus making it sensitive to moisture [85]. Meanwhile, cellulose is the most abundant renewable material, and it can exist in various forms upon modification. In packaging, cellulose can act as a filler or host polymer [86]. Chitosan is another attractive polymer that has been frequently investigated for antimicrobial packaging. Chitosan is generated from the deacetylation reaction of chitin. Chitosan possesses antimicrobial properties and, thus, can be used as a host and antimicrobial agent at the same time [47]. Table 2 shows some examples of bio-based polymers reported for antimicrobial packaging in recent years. They are being used with a variety of antimicrobial agents, both natural and synthetic, with various types of preparation methods involving solvent casting and encapsulation and being targeted for different types of microorganisms. In a way, bio-based polymers show a great potential in antimicrobial packaging; however, their relatively high cost compared to the traditional polymers somehow limits their applications on a larger scale.

## 3. Types of Antimicrobial Packaging

The antibacterial, antifungal, and antioxidant activities can be prompted by the main polymer used for packaging or by addition of numerous components from natural agents (bacteriocins, essential oils, natural extracts, etc.) to synthetic agents, both organic and inorganic (Ag, TiO_2_ nanoparticles, ZnO, synthetic antibiotics, etc.) [46].

**Figure 7:**
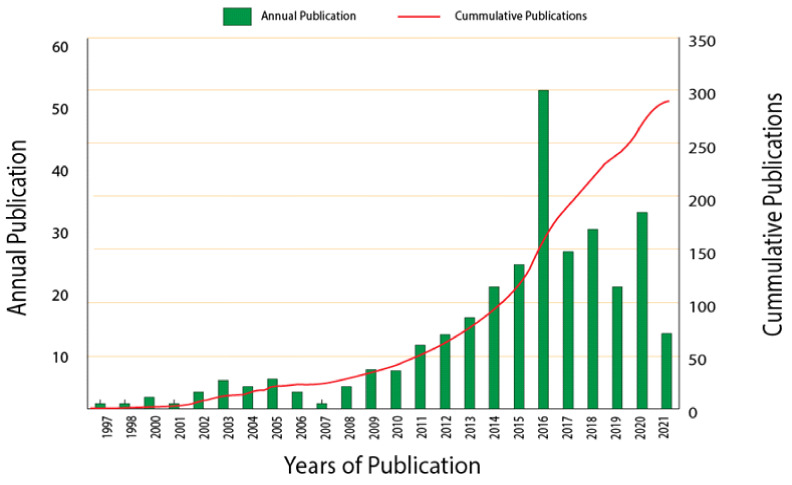
Annual and cumulative publications on antimicrobial packaging for various applications.

This review on antimicrobial packaging for various applications was supported with bibliometric analysis as a systematic approach. Data used in the present study were retrieved on 8 June 2021 from Scopus. Data from June 2021 onwards were not considered in this study for data consistency. Presently, to this writing, the keyword search analysis in Scopus on the query string (TITLE-ABS (“antimicrobial packaging”)) AND TITLE-ABS (food*) AND PUBYEAR < 2021 OR PUBDATETXT ((“January 2021” OR “February 2021” OR “March 2021” OR “April 2021” OR “May 2021”)) AND (EXCLUDE (PUBYEAR, 2022)) AND (LIMIT-TO (LANGUAGE, “English”)) resulted in 306 documents (Figure 7) wherein 195 were research articles, 56 were book chapters, 33 were review works, 18 were conference papers, and 4 were books (8 June 2020).

There are several forms of antimicrobial packaging, which are (1) addition of sachets/pads containing volatile antimicrobial agents into packages, (2) incorporation of volatile and non-volatile antimicrobial agents directly into polymers, (3) coating or adsorbing antimicrobials onto polymer surfaces, (4) immobilization of antimicrobials to polymers by ion or covalent linkages, and (5) use of polymers that are inherently antimicrobial [25,109]. 

Overall, the antimicrobial packaging strategy is classified into two groups, either direct or indirect contact between antimicrobial surface and the preserved food [46]. Table 3 briefly explains the definition, types, and function of the antimicrobial packaging strategies.

Firstly, the most common strategy is by having the antimicrobial sachet or pad with antimicrobial substance inside a sachet and added to the food packaging [46,110]. The antimicrobial compounds are released from the sachets into the headspace of packaging or to the surface of food products and subsequently inhibit the growth of food-borne pathogens [111]. The most popular antimicrobial agents for active packaging include nisin, chitosan, potassium sorbate, silver substituted zeolite, and essential oils [112].

Secondly is the inclusion or embedding of antimicrobials directly into the interior of the polymer films. In this method, the antimicrobial compounds are inside polymer films and introduced during the manufacturing process of these films [111]. The materials used in edible films should be Generally Recognized as Safe (GRAS) and may be eaten with food [113]. Thirdly is by covering the polymer surfaces with a layer of antimicrobial. The antimicrobial agents are coated onto the surfaces of the polymer films [98]. Then, the antimicrobial substance would either evaporate into the headspace or migrate into the food through diffusion [110].

The following antimicrobial packaging strategy is immobilization of antimicrobials in the polymers using ion or covalent linkages. This method needs (1) antimicrobial agents with functional groups that can be linked to the polymers and (2) antimicrobial compounds containing functional groups such as enzymes, peptides, and polyamines [98]. Lastly is the permanent existence of antimicrobial polymers. Some polymers used to construct films inherently have antimicrobial properties themselves [111]. For example, chitosan is categorized as an active food packaging material because of its inherent antimicrobial properties and capacity to carry various active components [114].

## 4. Performance of Antimicrobial Packaging 

As mentioned before, antimicrobial packaging is frequently prescribed as a combination of antimicrobial material and agents based on a specific matrix, which, in turn, leads to different types of packaging functions and uses. The review of correlation analysis between antimicrobial properties in terms of its use and advantages toward its application in food safety is presented in Table 4. What stands out in this table is the general pattern of each antimicrobial agent such as volatile gas form [115], silver compound [96], sanitizer and fungicide [116], plant extract [115], plant essential oil [115,117,118,119,120], enzyme [121], chitosan [122,123], bacteriocin [124,125], and inorganic nanoparticle [126,127] in controlling the growth of microorganisms, which, in turn, leads to indicators of prolonged shelf life of food, which is the essence of food safety. An important finding that emerged from the data was that antimicrobial agents can inhibit the growth of pathogenic microorganisms in food such as *Bacillus cereus*, *Escherichia coli*, *Listeria monocytogenes*, *Salmonella* spp., *and Staphylococcus aureus* [128] (Table 4). In addition, a further striking factor to emerge from the table is how antimicrobial material also shows a similar ability to protect from microbial growth by adding value by controlling the moisture migration and nutrient oxidation [115]. Taken together, these trends suggest that there is an association between antimicrobial properties toward superior food safety and longest shelf life.

**Figure 8:**
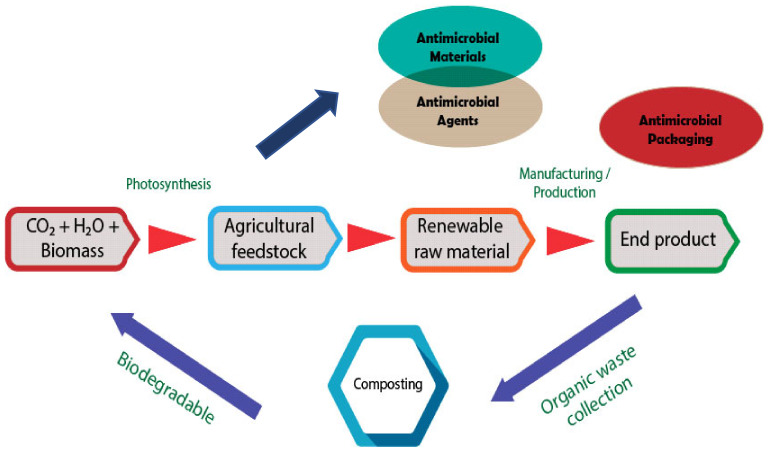
Interrelated antimicrobial packaging towards environmental impact.

The next part of the review was concerned with the performance of antimicrobial packaging towards environmental impact, as shown in Figure 8. Looking at Figure 8, it is apparent that the antimicrobial packaging showed a positive impact on the environment as well as in the ecosystem cycle. One concept that emerged during the extensive review was antimicrobial agents and material that normally come under renewable raw material provide added value in ensuring ecosystem sustainability. This is because, if antimicrobial packaging is made from a combination of antimicrobial agents and materials that are based on renewable raw materials, it may accelerate the biodegradation process and further stabilize the ecosystem balance. These results suggest that antimicrobial packaging is not only able to show the ability of controlling the growth of microorganisms and prolonging the shelf life of food, which is very important in food safety, but also has a positive effect on the environment [129].

**Table 4:** Use, Advantages, and Applications of Different Types of Antimicrobial Properties in Antimicrobial Packaging for Food Safety.

Type of Antimicrobial Packaging Properties	Example	Use	Commercial Product	Advantages	Application	Ref.
Antimicrobial Agents						
Volatile gas form	Chlorine dioxide, ethanol and sulfur dioxide	In sachets/pads that are attached to the internal part of the package	Maicrogarde^TM^, (BarrierSafe International Inc., Lake Forest, IL, USA)	Initiates the solid-state dry reaction, subsequently producing chlorine dioxide that diffuses throughout the package to inhibit microbial contamination and control odor Inhibit aerobic total viable count	Iceberg lettuce	[115]
			Ethicap^TM^ (Freund Corp., Tokyo, Japan)	Able to retard mold growth	Bakery and dried fish products	
Silver compound		Inhibit a wide range of microorganisms, bacteria, and mold by disrupting the microbial enzymes activities	Zeomic^TM^	Able to control the growth of gram-positive bacteria, gram-negative bacteria, and fungi	Chopping board, food packaging film, and glove and lunch box	[109]
			Aglon^R^ Novaron^R^ Cleanaid^TM^		Food packaging	
Sanitizer and fungicide			Mircoban^R^	Inhibited the growth of *S. Typhimurium* (ATCC 14028), *S. aureus* (ATCC 12598), *B. thermosphacta* (ATCC 11509), *B. subtilis* (ATCC 605), *E.coli* (ATCC 25922), *S. flexneri* (ATCC 12022), and several strains of *E.coli* O157:H7.	Packaging of meat	[116]
Plant extract	Wasabi extract		Wasapower^TM^	Volatile allyl isothiocyanate (AIT) play inhibits bacteria such as *E.coli*, *S.aureus*, fungi *A. niger*, and *P.italucum*.	Sushi products	[115]
Plant essential oils	Linalool, thymol, carvacrol, clove oil, cinnamaldehyde, basil essential oil			Inhibit microorganism growth through disturbance of the cytoplasmic membrane, disrupting the proton motive force, electron flow, active transport, and inhibition of protein synthesis.	Food packaging	[115]
	Grapefruit seed extract			Inhibit the growth of pathogenic bacteria such as *L. monocytogenes*, *E.coli*, and *S. Typhimurium*	Packaging ground beef	[118]
	Oregano essential oil and citral			Reduce the number of *E. coli*, *S. enteric*, and *L. monocytogenes* in salad	Packaging salad	[119]
	Allyl isothiocyanate (AIT)			Effective against *E. coli*	Ground meat patties	[120]
	Garlic oil			Inhibit the growth of microbial on sprout Effectively reduce the number of gram-positive and gram-negative bacteria Inhibit the growth *S. aureus* and *B. cereus*	Sprout	[117,130]
Enzyme	Lysozyme			Effective on *E. coli* O157:H7 and *S. typhimurium*	Beef patties	[121]
Chitosan				Reduce the number of *E. coli* and *S. aureus*	Food packaging,	[131]
				Reduce population of total aerobic count in pork	Vacuum packaging of refrigerated grilled pork	[132]
	Coated on plastic film, incorporated with 1% oregano oil and clove essential oil			Control cheese exhibiting *L. monocytogenes*, *S. aureus*, and *E. coli O157:H7*	Vacuum- sealed cheese	[123]
	Incorporated with nisin and Thymus kotschyanus essential oil			Inhibition level of *L. monocytogenes*, *E. coli*, *S. aureus*, and *S. Typhimurium*	Food packaging	[122]
Bacteriocin	Enterocins A and B			Control of proliferation of *L. monocytogenes*	Oyster and beef	[125]
	Pedicin			Reduction of *L. monocytogenes*	Raw chicken	[124]
Inorganic Nanoparticles	Titanium dioxide (TiO_2_)			Inactivate microorganism by oxidizing the polyunsaturated phospholipids’ component of the cell membrane Reduction of *E. coli* and *Pseudomonas* spp.	Food packaging	[126]
	Zinc oxide (ZnO)			Exhibited *E. coli* and *S. aureus*	Food packaging	[127]
Antimicrobial Material						
Biodegradable materials	Edible biopolymer		Protein, lipids, and polysaccharides	Protected from microbial growth, moisture migration, and nutrient oxidation	Packaging of nuts, candies, and fruits	[115]
	Food-grade additives		Plasticizers Colorant Flavors Emulsifiers antioxidants			

**Figure 9:**
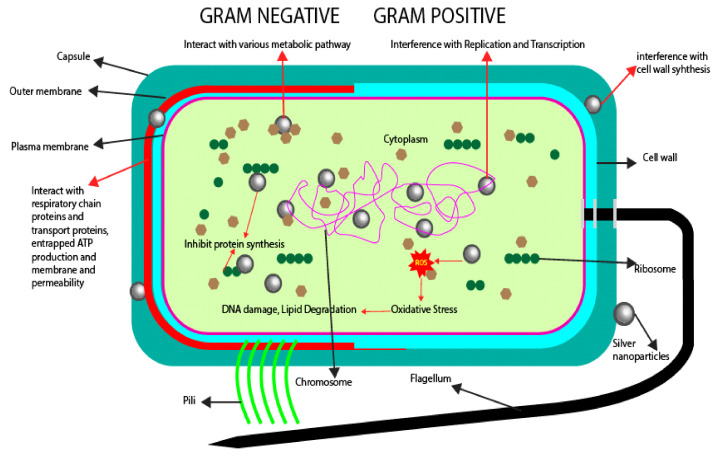
Possible mechanism of silver nanoparticles towards microbes.

Many studies have been conducted on the mechanism of silver nanoparticles as an active packaging ingredient in packaging [53,55,58,133,134,135,136,137,138,139]. Figure 9 shows the possible mechanism of silver nanoparticles’ action in the antimicrobial food packaging. The mechanism of the AgNPs were said to impede the cell wall synthesis in the cell [139]. Daneshniya et al. (2020) [140] reported that the AgNPs in the range of 1–10 nm in size attached to the cell membrane and disrupted the membrane functions such as permeability and respiration. The silver nanoparticles were believed to be penetrating the bacteria cell and causing further wreckage by associating with the thiol groups from the respiratory chain proteins and transport proteins such as DNA, glutathione (GSH), and thioredoxin, which led to hindering their functions [137]. The damage towards the thiol groups was also stated to be due to the release of silver ions (Ag+) from the silver nanoparticles that are very reactive when they are reacted with the cell membrane that was negatively charged [41]. The reaction resulted in further involvement in the bactericidal effect of silver nanoparticles and led to cell death [135,137]. However, the specific action of silver nanoparticles’ mechanism was still unclear throughout these extensive studies.

All organic and inorganic compounds that were widely studied in the past research such as chitosan, chitin, titanium oxide (TiO_2),_ and copper (Cu) have shown great antimicrobial effects toward the bacteria, microorganisms, and enzymes, as mentioned in Table 5. Table 5 depicts the widely used material of antimicrobial agents in producing the antimicrobial food packaging. 

## 5. Issues Related to Antimicrobial Packaging

Application of antimicrobial packaging systems based on biopolymers incorporated with different bioactive agents possesses immense potential for improving the food quality and safety along with a possible increment in shelf life. As mentioned earlier, a variety of bioactive substances, both synthetic and natural, such as essential oils, antimicrobial peptides, enzymes, etc., have been investigated and applied in antimicrobial packaging systems. Several investigations on the subject have indicated the potential of antimicrobial packaging systems in effectively inhibiting the targeted spoilage microorganisms, employing a suitable combination of biopolymer and a bioactive compound to produce an antimicrobial film [166]. 

Despite all the above advantages of antimicrobial packaging, there are some challenges and limitations, which should be discussed and overcome. One of the main challenges is health issues and risks regarding the safety and migration of nanoparticles of antimicrobial agents. The possibility of inhalation by the respiratory system, skin penetration through skin nodes, and unintentional migration and ingestion of nanoparticles by the digestive system might badly affect human health.

### 5.1. Safety Issues

**Figure 10:**
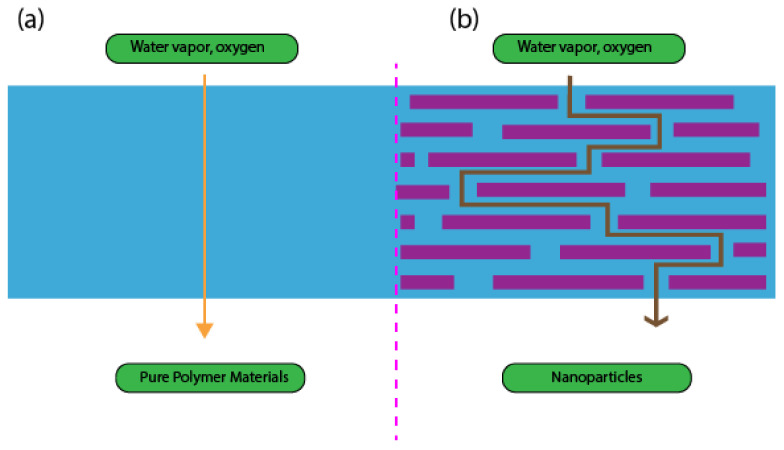
Water vapor and oxygen passing through (**a**) pure polymer materials and (**b**) nanoparticles.

Numerous studies have found that nanoparticles of antimicrobial agents are effectively proven in enhancing the barrier, mechanical, and antimicrobial properties of antimicrobial packaging when appropriate amounts of antimicrobial agents are incorporated into packaging materials. Figure 10 illustrates how nanoparticles of antimicrobial agents can improve the barrier properties as compared with pure polymer materials. Nanoparticle and pure polymer matrix properties are among the most important factors that determine the properties of the resulting composite. For food packaging applications, nanocomposites that have been studied the most are clay and polymer nanocomposites, while bio-based polymers that have been studied the most are PLA. These nanomaterials will intensify the water and serve as moisture-repellent properties of food packaging materials. 

However, there are a few limitations and issues that need to be reconsidered. In terms of migration, nanoparticles are susceptible to migrate from packaging into the food, which depend on nanomaterial characteristics such as size, concentration, shape, and dispersion. Other than those, there are environmental factors (temperature, mechanical stress), food condition (composition and pH), polymer properties (viscosity and structure), and contact duration. These will bring limitations and potentially result in adverse health effects. It has been reported that some nanoparticles can cause intracellular damage, pulmonary inflammation, and vascular diseases [167]. Thus, a detailed toxicological analysis is needed to explain the risks. 

There are three types of migrations of substances into food: (1) overall migration limit (OML), which evaluates the total weight of extracted substances, which is non-specific and above the limit that is allowed to be penetrate into food; (2) specific migration limit, which measures the concentration of the material-specific restricted substances based on their toxicological risks using advanced detecting assays; and (3) maximum permitted quantity (QM), which measures the maximum level of the residual given substance that can migrate from a material into foodstuffs or simulants. To ensure the overall quality of the plastics, the overall migration to a food of all substances together may not exceed the OML, which, for polymers of about 60 mg/kg of food (or food simulant) or 10 mg/dm2 of the contact material, will usually be used for inertness of the substances. Regulation No. (EU) 10/2011 from Plastics Regulation and No. (EU) 2016/1416 from the European Commission-published Commission Regulation ensure the safety of plastic materials with the use of migration limits, which specify the maximum amount of substances allowed to migrate to food. They impose the permitted value of 5 to 25 mg zinc per kg food (25 to 5 mg/kg food) for food contact items based on the SML consideration. Furthermore, 40 mg/day of zinc daily consumption for the human body is the restricted amount level for food contact materials by the National Institutes of Health [168]. A nanocomposite containing 0.5 g/L 0.5 g/L ZnO NPs is the permitted level of value of migration [169]. There was a study conducted by Bumbudsanpharoke et al. who experimented and discovered the migration of Zn^2+^ from LDPE-ZnO nanocomposite films, in which the level of migrated Zn^2+^ (3.5 mg L^−^^1^) was considered safe for human health due to a lower value than the specific migration limit provided by European Plastics Regulation (EU No. 10/2011) [170]. Examples for Specific Migration Limits are Polycyclic Aromatic Hydrocarbons (PAH) from carbon black and Bisphenol A (BPA) from polycarbonate plastic, most of the time known to be carcinogens. However, except in some cases, the level of migrated Zn^2+^ increased despite the migrated level being lower than the maximum migration limit based on the National Institutes of Health for food contact materials with the existence of essential oil in the nanocomposite [171].

### 5.2. Production Cost

Antimicrobials of nanoparticles are diversely used in packaging materials due to their advanced properties for industrial purposes. Most common uses of antimicrobial properties are Ag, TiO_2_, and ZnO of antimicrobial packaging systems. These types of nanoparticles are used in the lab and the production cost could be considered as way more affordable than the production cost for the real industry, in which the production costs required are 10 times more to be useful as the original one. The prices of antimicrobial agents are way more expensive in industrial scale, and scaling up the packaging for nanocomposites demands cutting-edge technologies, which may amplify the final cost, thereby reducing the market acceptability [172,173]. Apart from that, antimicrobial agents are frequently developed for a specific food and do not provide the same results with other types of food; thus, the price will be more expensive to buy several antimicrobial agents for several types of food.

### 5.3. Strong Aroma, Flavor, and Color

Essential oils of natural antimicrobial agents such as carvacrol, ginger/garlic oil, linalool, clove oil, thymol, basil, and cinnamaldehyde possess a high intensity of off-flavors. These types of essential oils have high antibacterial properties but have a strong smell and flavor, which inhibits the original flavor of the food, which represents the critical challenges for the food industry. Moreover, they carry a striking color. It was mentioned by [174] Bhullar et al. in 2015 that around 85–99% of essential oils contain phenolic and hydrophilic volatile terpenoids, which cause a generation of intense reddish color to the films. Furthermore, they have a sharp flavor, which restricts their applications in the food packaging industry constituents [174].

## 6. Conclusions and Future Perspective

The review presented here summarized comprehensive available information on the recent development of antimicrobial packaging, especially in food packaging industries. This review introduced a brief background on the concept of antimicrobial packaging and their principles, followed by the main components of the antimicrobial packaging composition. The discussions were narrowly focused into the types of antimicrobial packaging, the applications, the implementations of recent discoveries, and strategies aiming to curb microbial growth through innovative antimicrobial packaging. Among the demonstrated potential applications, their massive use in food packaging has received considerable interest compared to others. The reviewed research work from the literature offers evidence in favor of antimicrobial packaging use to control food quality over targeted perishable products, generally, and the current plan to execute the mass production of antimicrobial packaging in real food systems, specifically. The antimicrobial packaging synergistically made of selected green polymers incorporated with certain chemical agents, natural agents, or probiotics have been shown to be effective to address issues on antimicrobial activity and plastic pollution towards sustainable development. The strong ground supported by the regulatory authorities, the commitments from industry players, and the growing public awareness are pacing the anticipation toward the use of antimicrobial packaging. The strategies of hybridizing those inexpensive, abundant natural polymers with functional additives will enhance the polymeric properties in order to satisfy the stringent requirement set by the packaging industry. At the time of writing, countless efforts were made to accelerate the mass production of antimicrobial packaging throughout technological advancement. However, there are a few challenges that are faced during the replacement transition from conventional petroleum-based plastic packaging towards antimicrobial packaging materials. The consideration towards the suitably formulated components between various antimicrobial agents and polymeric matrices needs to be really understood. For instance, some of the potential antimicrobial agents such as essential oils might also experience a high loss rate due to rapid volatilization due to several causes. Oxidative and polymerization processes may result in a loss of quality and pharmacological properties. A slow and sustained release of the essential oils will be useful to maintain food quality due to the presence of a high concentration of essential oils trapped in the packaging. Further, in-depth research is required to limit volatile loss and to sustain the durability and efficiency of the fabricated antimicrobial packaging materials at their optimum.

The advanced technology offered in the innovative antimicrobial packaging also has countered the resistance phenomenon in microbes to conventional processing technologies. Despite the excellent antimicrobial activity in controlling the microbial contamination by reducing the growth rate and extending the lag period of targeted foodborne pathogens, the depth of evaluation of the migration of active antimicrobial agents throughout the packaging needs to be accentuated. The importance of preventing the migration of active substances throughout the packaging materials has drawn attention from consumers and regulatory authorities, in regard to human health due to the fact that some can cause irritation due to cytotoxic effects while others can be allergens. Migration of undesirable substances must be strictly under the limit established by regulations to protect the safety of the consumers. For nanoparticles-embedded packaging, the specific toxicological tests are of the utmost necessity for future studies to clarify that prolonged consumption of packaged food from these innovative packaging materials are safe to humans, without long-term side effects. The application of nanoparticles into the food packaging needs to have a concise guide and should be carefully assessed prior to being available on the market. Despite having many outstanding properties and a realm of possibilities for antimicrobial agents for the packaging industry for retarding microbial growth and improving the shelf life of foods, more comprehensive research is still a requirement, considering the above-mentioned limitations. Otherwise, the advantages of a prolonged shelf life may come at the expense of major unforeseen health repercussions.

Apart from that, the possible incoming threats to both terrestrial and aquatic ecosystems and the adverse effects of these antimicrobial substances-embedded packaging to long-term environmental impact should be considered. The disposal issue regarding the probability of the packaging containing nanoparticles and their subsequent breakdown, which could result in the release of unstable forms of chemical compounds into our natural ecosystems, should be highlighted. More future research should be focused on fully biodegradable polymers such as blends of starch and others for their high-efficiency usage in food packaging. Biopolymers are prominent candidates to be modified or combined with an antimicrobial substance to develop the antimicrobial systems with applications in several fields and in good directions to reach these goals. 

## Figures and Tables

**Table 1:** Petroleum-based polymers for antimicrobial packaging.

Host Polymer	Antimicrobial Agent	Preparation Method	Targeted Organism	Ref.
PVC	Ag-NP	Solvent casting	*B. subtilis*, *A. niger*, and *F. solani*	[66]
PVC	PHE-Zn	Solvent casting	*E. coli* and *S. aureus*	[67]
PVC	Orange essential oil	Solvent casting	*E. coli* and *S. aureus*	[68]
PET	Ag-NP	Melt blending	*E. coli* and *Z. Bailii*	[71]
PET	LDH-p-hydroxybenzoate	Coating	*Salmonella* spp. and *C. jejuni*	[69]
PET	ZnO, TiO_2_	Melt blending	*-*	[72]
LDPE	ZnO	Melt blending	*E. coli*	[73]
LDPE	Ag-NP	Melt blending	*E. coli*, *S. aureus*, *E. faecalis*, and *Salmonella enterica*	[74]
LDPE	Thymol	Solvent casting	*E. coli* and *Salmonella enterica*	[75]
PE	Carvacrol and menthol	Coating	*E. coli*, *S. aureus*, *L. innocua*, and *S. cervicea*	[76]
PP	Sorbic acid	Extrusion molding	*E. coli* and *S. aureus*	[77]
PP	Oregano EO	Melt blending	*B. thermosphacta*	[78]
PP	Carvacrol	Melt compounding	*E. coli and A. alternata*	[79]
PS	GO-p(VBC)	Solvent casting	*B. cereus*, *P. aeruginosa*, and *fungus candida*	[80]
PS	ZnO-NP CaCO_3_-NP TiO_2_-NP	Encapsulation	*S. aureus*, *P. aeruginosa*, *C. albicans*, and *A. niger*	[81]

Nanoparticle (NP); Pentaerythritol p-hydroxybenzoate ester-based zinc metal alkoxides (PHE-Zn); Layered double hydroxide para-hydroxybenzoate (LDH-p-hydroxybenzoate); Essential Oil (EO); graphene oxide/poly(4-vinylbenzyl chloride), GP(VBC).

**Table 2:** Antimicrobial packaging systems utilizing bio-based polymers.

Host Polymer	Antimicrobial Agent	Preparation Method	Targeted Organism	Ref.
PLA	ZnO, MgO, TiO_2_	Solvent casting	*E. coli*	[70]
PLA	ZnO	Solvent casting	*E. coli* and *L. monocytogenes*	[87]
PLA	TV-EO, EEP	Solvent casting	*S. aureus* and *Penicillium* sp.	[88]
Starch	Nisin and Natamycin	Solvent casting	*B. cereus* and *A. niger*	[89]
Starch	Ferulic acid, Cinnamic acid	Melt blending	*E. coli* and *L. innocua*	[90]
Starch	Carvacrol, montmorillonite	Solvent casting	*E. Coli*	[62]
Carrageenan	Orange essential oil, Trehalose	Solvent casting	*S. aureus*, *E. coli* and *C. albicans*	[91]
κ-Carrageenan	Olive leaves extract	Solvent casting	*E. coli*	[92]
κ-Carrageenan	CuS-NP	Solvent casting	*S. aureus* and *E. coli*	[93]
Nanocellulose	Nisin	Solvent casting	*L. monocytogenes*	[94]
Carboxymethyl Cellulose	Curcumin, Zinc Oxide	Solvent casting	*L. monocytogenes* and *E. coli*	[86]
Nanocellulose	Anthocyanin, Oregano essential oil	Solvent casting	*L. monocytogenes* and *E. coli*	[95]
Gelatin	Bacteriophages	Solvent casting	*S. aureus*	[96]
Gelatin	Curcumin	Solvent casting	*E. coli* and *L. monocytogenes*	[97]
Gelatin	Pomegranate peel powder	Solvent casting	*S. aureus*, *L. monocytogenes* and *E. coli*	[98]
Pectin	Copaiba oil	Solvent casting	*S. aureus* and *E. coli*	[99]
Pectin	Ag-NP	Solvent casting	*E.coli* and *Salmonella* Typhimurium	[100]
Pectin-Alginate	Carvacrol	Encapsulation	*E. coli*	[101]
Alginate	Sulphur-NP	Solvent casting	*E. coli* and *L. monocytogenes*	[102]
Alginate-Chitosan	ZnO-NP	Coating	*-*	[103]
Alginate-Chitosan	Nisin	Encapsulation	*L. monocytogenes*	[104]
Chitosan-Starch	Grapefruit seed extract	Solvent casting	*A. niger*	[85]
Chitosan	Proanthocyanidins	Solvent casting	*E. coli*, *Salmonella*, *S. aureus*, and *L. monocytogenes*	[105]
Chitosan-Agar	Ag-NP	Solvent casting	*P*. *aeruginosa*, *E*. *coli*, and *S*. *aureus*	[106]
Agar	Ag-NP	Solvent casting	*A. hydrophilla*	[107]
Agar- Carboxymethyl Cellulose	Ag-MMT	Solvent casting	*B. subtilis* and *E. coli*	[108]

*Thymus vulgaris* essential oil (TV-EOs); ethanolic extract of Mediterranean propolis (EEP); Silver modified montmorillonite (Ag-MMT).

**Table 3:** Description of antimicrobial packaging strategies.

Strategies	Definition	Types	Function
Antimicrobial sachet or pad	The most common type of antimicrobial packaging. The sachets or pads that contain antimicrobial packaging are attached, enclosed, or loose in the interior of a package.	Three types of antimicrobial agents added in the sachets or pads are oxygen absorbers, moisture absorbers, and ethanol vapor generators.	To prevent oxidation, inhibit growth of molds, and lower water activity.
Direct integration in polymer	Any polymer used for packaging is incorporated with antimicrobial agents.	Edible films incorporated with nisin, lysozymes, antimicrobial enzymes (lactoferrin and lactoperoxidase), antimicrobial peptides (magainins, cecropins, natural phenols, antioxidants), metals (copper), and zeolites substituted by 1–3% silver incorporated into polyethylene, polypropylene, nylon, and butadiene styrene.	To disrupt the enzymatic activity of microbial cells and to prevent surface growth in packages.
Antimicrobial coating	Applying antimicrobial coatings on the polymer surfaces such as films, wax paper, and cellulose casing.	Waxes, fungicides, sorbic acid, polyethylene films coated with nisin/methylcellulose, poultry coated with nisin/zinc	For wrapping or packaging materials.
Immobilization of antimicrobials to polymers by ionic or covalent linkages	Ionic and covalent immobilization of antimicrobials onto polymers with the presence of functional groups and spacer molecules that link antimicrobial agents to polymers surfaces.	Antimicrobial agents with functional groups are peptides, enzymes, polyamines, and organic acids, whereas antimicrobial compounds with functional groups are enzymes, peptides, polyamines ethylene vinyl acetate, ethylene methyl acrylate, ethylene acrylic acid, ethylene methacrylic acid, ionomer, nylon, polystyrene, etc.	To reduce antimicrobial activity per unit area such as in proteins and peptides due to change in conformation and denaturation by solvents.
Inherently antimicrobial polymer	Cationic polymers that are inherently antimicrobial, and physical modification of polymers were used in films and coatings.	Chitosan and poly-1-lysine polymers films and coatings, polyamide films treated with UV irradiation.	It acts as a barrier between the nutrients contained and microorganisms to protect them from fungal degradation.

**Table 5:** Advantages and disadvantages of widely used materials of antimicrobial agent for food packaging industries.

Antimicrobial Agent	Advantages	Disadvantages
Silver	Act as a catalyst instead of chemically reacted with microorganisms in their destruction, and microorganism cannot resist them [141]. Eliminate the risk of genetic mutations of microorganisms due to direct use of toxins [141]. Can be combined with both degradable and nondegradable biomaterials, resulting in improving the permeability of the film, quality of the product, and mechanical properties of the coating [54,141]. Stable in very high temperature compared to other compounds [142].	Can cause particles to migrate from packaging to the food if it is used at a high level [143,144].
Titanium oxide	High stability, extensive range of antibiosis [145]. Biologically inactive, demonstrates quite low toxicity, thus low risk to human [146]. Shows no absorption or tissue storage of TiO_2_ and no hazardous effects for occupational workers and public health [147].	All molecular sizes of TiO_2_ and crystal forms (anatase and rutile) might cause phototoxicity due to reactive species (ROS) under UV radiations [147]. Reactive oxygen species (ROS), such as hydrogen peroxide (H_2_O_2_), hydroxyl radicals, and superoxide lead, which can lead to the oxidative stress pathway. This is one of the ways in which TiO_2_ and Ag NPs exert their toxic effects and interrupt the life cycle of Drosophila through the ROS generation enhancement and DNA damage that led to related adverse consequences [148].
Copper	Inhibits or declines bacteria, viruses, and fungi growth [141] Increases the film thermal stability and mechanical properties [141] Can inhibit survival of microorganisms [149]	Toxic, especially at the nanometre dimension [141] Nanoscale size Cu increases their reaction since the surface atoms are increased, which make them highly reactive sites, causing severe antimicrobial behavior and toxicity [141,150]
Chitosan	Water-soluble cationic polymer due to positive charge on its amino groups [151]. Polycationic, non-toxic, biocompatible, and biodegradable [152,153,154,155]. Soluble in dilute acids in pH less than 6.0–6.5, for example, acetic acid, formic acid, lactic acid, and HCI [156] Good mechanical properties and can be consumed along with the product in the package [157,158]	Insoluble at neutral and higher pH due to the D-glucosamine [156,159]
Chitin	Renewable, biocompatible, biodegradable, and non-toxic compounds [160,161,162]. Abundant [151] Antioxidant [163]	Highly hydrophobic, thus insoluble in water and even organic solvent [151]
Lysozyme	Naturally present in avian eggs and mammalian milk [160] Cost effective [164] Showed high activity towards Gram-negative bacteria and moderately effective against Gram-positive bacteria [165]	Showed no action towards yeasts or fungi [165]

## Data Availability

The data that support the findings of this study are available on request from the corresponding author.
